# A second-generation anchored genetic linkage map of the tammar wallaby (*Macropus eugenii*)

**DOI:** 10.1186/1471-2156-12-72

**Published:** 2011-08-19

**Authors:** Chenwei Wang, Lee Webley, Ke-jun Wei, Matthew J Wakefield, Hardip R Patel, Janine E Deakin, Amber Alsop, Jennifer A Marshall Graves, Desmond W Cooper, Frank W Nicholas, Kyall R Zenger

**Affiliations:** 1 Reprogen, Faculty of Veterinary Science, The University of Sydney, Sydney, NSW 2006, Australia; 2 Australian Research Council Centre of Excellence for Kangaroo Genomics; 3 Research School of Biology, The Australian National University, Canberra, ACT 0200, Australia; 4 Bioinformatics Division, The Walter and Eliza Hall Institute of Medical Research, Parkville, VIC 3052, Australia; 5 School of Biological, Earth and Environmental Sciences, The University of New South Wales, Sydney, NSW 2052, Australia; 6 School of Marine & Tropical Biology, James Cook University, Townsville, QLD 4811, Australia

## Abstract

**Background:**

The tammar wallaby, *Macropus eugenii*, a small kangaroo used for decades for studies of reproduction and metabolism, is the model Australian marsupial for genome sequencing and genetic investigations. The production of a more comprehensive cytogenetically-anchored genetic linkage map will significantly contribute to the deciphering of the tammar wallaby genome. It has great value as a resource to identify novel genes and for comparative studies, and is vital for the ongoing genome sequence assembly and gene ordering in this species.

**Results:**

A second-generation anchored tammar wallaby genetic linkage map has been constructed based on a total of 148 loci. The linkage map contains the original 64 loci included in the first-generation map, plus an additional 84 microsatellite loci that were chosen specifically to increase coverage and assist with the anchoring and orientation of linkage groups to chromosomes. These additional loci were derived from (a) sequenced BAC clones that had been previously mapped to tammar wallaby chromosomes by fluorescence *in situ *hybridization (FISH), (b) End sequence from BACs subsequently FISH-mapped to tammar wallaby chromosomes, and (c) tammar wallaby genes orthologous to opossum genes predicted to fill gaps in the tammar wallaby linkage map as well as three X-linked markers from a published study. Based on these 148 loci, eight linkage groups were formed. These linkage groups were assigned (via FISH-mapped markers) to all seven autosomes and the X chromosome. The sex-pooled map size is 1402.4 cM, which is estimated to provide 82.6% total coverage of the genome, with an average interval distance of 10.9 cM between adjacent markers. The overall ratio of female/male map length is 0.84, which is comparable to the ratio of 0.78 obtained for the first-generation map.

**Conclusions:**

Construction of this second-generation genetic linkage map is a significant step towards complete coverage of the tammar wallaby genome and considerably extends that of the first-generation map. It will be a valuable resource for ongoing tammar wallaby genetic research and assembling the genome sequence. The sex-pooled map is available online at http://compldb.angis.org.au/.

## Background

There are about 270 marsupial species, which are distributed throughout Australasia, and in the Americas. Marsupials are interesting not only because of their unique biology, but also because of their distinctive evolutionary position between birds and eutherian mammals, so that comparisons provide valuable insights into sex determination, lactation, immunology, cancer, genomics and evolution [1]. Two model marsupial species have been intensively studied both biologically and genetically, the South American grey short-tailed opossum (*Monodelphis domestica*) and the Australian tammar wallaby (*Macropus eugenii*). The genomes of both species have been sequenced, with the opossum genome sequenced at 7-fold coverage [2], and the tammar wallaby genome sequenced at 2-fold coverage [3].

The tammar wallaby genome is divided into eight pairs of large chromosomes (seven pairs of autosomes and a pair of sex chromosomes: XX for female and XY for male). The 2n = 16 karyotype is readily comparable with the conserved marsupial ancestral karyotype of 2n = 14 [4]. Chromosome painting studies [5,6] have revealed highly conserved chromosome regions amongst many marsupial species, even between distantly related groups. This conservation means that the genome assembly of the tammar wallaby will accelerate studies of other marsupial genomes.

A genome-wide genetic linkage map with anchored genetic markers corresponding to coding genes is an important genomics tool, providing a valuable resource for gene/QTL localisations and comparative genomic investigations. Furthermore, it provides a major independent source of information for robust genome sequence assembly. A first-generation tammar wallaby linkage map was constructed by Zenger et al. [7] with 64 markers covering a total length of 828 cM, with average distance between adjacent markers of 16.2 cM. This first-generation linkage map was based primarily on a small number of unanchored type II genetic markers (e.g., anonymous loci with limited flanking sequence) and hence this map has limited utility in comprehensive comparative genomics or sequence assembly investigations.

Given the limited genome coverage and genetic marker type in the first-generation tammar wallaby linkage map, there is an urgent need to extend this resource to include genome-wide anchored type I (i.e., coding genes) genetic markers. Here, we present a second-generation tammar wallaby linkage map containing more than twice the number of genetic markers in the first-generation map. Importantly, particular care was taken to include physically anchored and type I genetic markers (chosen on the basis of FISH mapping) to provide coverage of most regions of all chromosomes, for increased utility in direct comparative mapping investigations. It is anticipated that this resource will be highly useful in ongoing genome investigations and marsupial sequence assemblies.

## Methods

### Linkage mapping reference families

Inter-subspecies crosses between island populations of tammar wallabies contributed the majority of genetic variation needed for constructing the map [8]. The use of sub-species in this experimental design increases the likelihood of producing informative heterozygous genotypes in parental individuals. This study utilised the same well-established and validated Garden Island (GI) and Kangaroo Island (KI) hybrid backcross pedigrees/DNA used to create the first-generation genetic linkage map [7]. This research was performed purely on extracted DNA samples from previous studies where appropriate animal ethics approval had been granted. All pedigrees are of a three-generational design (G0, G1 and G2) allowing for the phasing of G2 genotypes. All three generations were genotyped and independently compared within each pedigree. Both G1 hybrid males and G1 hybrid females were used as parents of G2 animals, so that sex-specific maps could be produced. In total, there are 4 male pedigrees generating 104 G2 offspring, and 21 female pedigrees producing 127 G2 offspring [7]. Only those families with genotypic data from all three generations were used in this study. Thus an additional 121 offspring from phase-unknown males (no G0 grandparent genotypes available) used in the previous study [7] were excluded from the present study. The informative meiosis numbers for the male and female pedigrees at each locus are presented in Tables 1, 2 and 3.

**Table 1:** Set 1 markers: BAC clone, chromosome location and primer sequences

**Marker Name**^**a**^	BAC clone	Chr	Arm	Band	Informative meioses		**MgCl**_**2 **_**(mM)**	Primer sequences
							
					M	F		
KJW105	MeVIA123J11	1	p	2	3^d^	64	1.5	F: TAAAAGCGCTGGGTGATTTC R: GGTTATCACATTTGGAAACAAAGC
KJW117	MeVIA72C1	X	q	3	0	89	1.5	F: AAGAGGTGAGTTGGGACACG R: TGGCCTGGGATTAAAAGTCA
KJW122	MeVIA78K19	1	q	3	61	106	1.5	F: GCAGCTTCAGAAATGCAAAC R: GGTTCTGCAAACTCCAGTGAG
KJW174^b^	NA^c^	NA^c^	NA^c^	NA^c^	97	89	2.5	F: ACGTGTATGTGTAAGTGTGTATGT R: CCTGGCACCTAGATAAAGCA
KJW175	MeVIAP6	7	p	3	94	122	1.5	F: CACAGAAACTTGGGGAAAGC R: TCTTCCTGATGGGATGAAGG
KJW186	MeVIA49J18	2	q	1	93	115	1.5	F: GCTTTTTCAAAGGGACATTTTC R: CGCACTCTTTCAAGGGACTG
KJW192	MeVIA142H21	5	q	2	45	94	1.5	F: ATGGGGAAACTGAGGCAGAG R: AGTTGGAACCACCTCCACTG
KJW208^b^	NA^c^	NA^c^	NA^c^	NA^c^	89	104	1.5	F: ACTGAGACAATGCCTTTCC R: TTCCTGCCTTCTTTACTCC
KJW220	MeVIA115N20	7	q	3	7^d^	58	1.5	F: TATCTCATGGGGAGGAGGTG R: CCAGGTCAAACACAGTGAGC
KJW221	MeVIAP6	7	p	3	94	125	1.5	F: CAGCAGAAGAGGGGAGACTG R: CACAGAAACTTGGGGAAAGC
KJW243	MeVIA35G12	X	q	2	0	102	1.5	F: ATTGGTGAATGGCAAATGAG R: CCTTCTTCCTTTGTCCTGATG
KJW258	MeVIAE9	5	p	2	90	110	1.5	F: TGTGAGGCAAAGAAATTCCAG R: ATCAGCCCTGGGGATAGATG
KJW281	MeVIA15A6	X	q	2	0	100	2.5	F: GACCAGGTTGTTGGGTCAAG R: CATTCAGGACAGGTAGGTAGGG
KJW285^b^	NA^c^	NA^c^	NA^c^	NA^c^	100	118	1.5	F: ATCCAAGATGATGGCCTGAG R: AGAGAGCTCGGTGGCATTAC

^a ^Set 1 marker names comprise "KJW" followed by an identification number, e.g. KJW174.

^b ^These three markers could not be located on any of the 44 BACs. This may reflect failure to optimize the PCR to a working condition in the BAC, or an error in identifying BAC clone during FISH-mapping BACs or screening for AC repeats.

^c ^NA = not available

^d ^Meiosis number less than 19, and not included in the map construction.

**Table 2:** Set 2 markers: chromosome location and primer sequences

**Marker Name**^**a**^	Physical location (ChrArmBand)	Informative meioses		**MgCl**_**2 **_**(mM)**	Primer sequences
				
		M	F		
MeKba170K8-13193	2q2	77	102	2.5	F: CATACCTTCTCTTGTCTTGTGGC R: TTCATATTGGAGGAGGATTAGC
MeKba175L17-104075	3q2	93	121	1.5	F: CCATCACATACCACACACGC R: TTGGCTATAATTGTGGTCAGC
MeKba182A19-136367	3q2	95	81	1.5	F: TGTTAATGTATCATCATCACTCTCC R: GAAGGAACAATGACAGAACTTAGC
MeKba191O7-75495	1q3	82	85	2.5	F: TATGCATCGAGTGCCTGC R: AGCCTTCCTTGCCATTAACC
MeKba206L23-25864	2q3	84	111	1.5	F: GCCGTGAGCACTCTGTCC R: CCATCCTCAATCTCCTCTCC
MeKba273F23-57082	1q2.2 (1q5)^b^	80	88	2.5	F: GTGCCACTGTAGTCCACCTG R: CTGGTTCTGGTCTCTGGAGC
MeKba281G14-77691	6q3 (1q3-4)^b^	51	123	1.5	F: ATCGACAGCCTCTCCAACC R: GGCAATGAGATGAGATGTGC
MeKba282N22-44716	1q4-5	95	107	2.5	F: GGTCAGACACGCACAACC R: CAGAATTGGCACCTAGATATTCC
MeKba337B13-128470	2q3	68	75	1.5	F: TTAATGAATTCCAATGGCTACC R: GAGTACATTCCAGGCATAGTGG
MeKba389E8-21049	2q2	72	86	2.5	F: ATCTAATGATAGCCACCTCTGG R: TGACTGACAACTTAGCCTGCC
Mekba458L18-116052	2q3	81	117	1.5	F: CTCTGGCTCAGGTCCTTCC R: TCTATTCTCCTGTGATCCTATGC
MeKba472N21-102181	3q1	89	84	1.5	F: AGGAGGACTGGAGGAATTAGG R: GAGAAGTGAGCCTGGACAGC
MeKba494M2-50126	Xq3	0	91	2.5	F: GTCGCACAGCTGGTTAAGG R: GCATTCTTATTGGAACTGTGACC
MeKba510M2-126695	Xq2	0	71	2.5	F: ACCACACAGACACATGCACC R: GAATAGTCCACCACCACTCTAGG
MeKba510M2-pseudo^c^	NA^d^	61	88	2.5	F: ACCACACAGACACATGCACC R: GAATAGTCCACCACCACTCTAGG
MeKba526C2-33878	1q5	85	125	1.5	F: GGTTGCATTCACTGGTCTAGG R: GGTTGCATTCACTGGTCTAGG
MeKba528O13-122762	3q2.3	0^e^	27	2.5	F: CACTGTGCTATCTGCTGAAGG R: GATGGCGTGGTCTTCTTAGG
MeKba598C23-22378	Xq2	0	99	1.5	F: CCATTGCTACTACCTTCAGCC R: GGTGAGGTGATATTCTGTCTTGG
MeKba60J17-8783	3q2.3	93	107	2.5	F: ACATTCTTGCCAGGCTCACC R: AGTGGAGGCATCTCAAGGC
MeVIA121C8-17366	Xq3	0	83	1.5	F: TTTCTCAGCCACACCTCTCC R: ATATGCCCCAAAAGGAGCAC
MeVIA1A16-106	2q5	1^e^	42	2.5	F: AGCTCATTGTGAGAACTCGG R: TGTAAGTTAGCATGGTGAAGAGC
MeVIA1B23-391	4q2.3	89	107	2.5	F: TGAAGGCTTGACTTCCTTCC R: TTCATCATGTCTGTAGCCTTAGC
MeVIA1B5-253	4p2.1	91	106	2.5	F: CAGTTACCTGGTGATGACTTGC R: GGAATTAGCTGTTCAGGAATAATTAGG
MeVIA1G15-185	5q1	97	105	2.5	F: CCGCCTATCCTCAATAACTGC R: GAATAACAACAGACACACACACG
MeVIA1G3-226	1q1 (7p3)^b^	61	100	2.5	F: TCTTCACATTAGAGAACAGAACAGG R: GACACCTCTGCTCCACACC
MeVIA125B16-39822	NA^d^	92	110	1.5	F: CTCTTCCCCACTCCCCTATC R: CCCATGGATTGGAGGATTAC
MeVIA1L6-488	1q3	99	123	1.5	F: ATTCATCCATCCATCCATCC R: AAGGTTGTTAAGTGGCAGAGC
MeVIA2C3-167	3q	56	91	2.5	F: CATGCCAACTCTCTATGTATTGG R: CAGATGAGGTATGGTCAACAGG
MeVIA2J14-517	4p3.3	43	93	1.5	F: GAGGATGGTGATGAAGCAGG R: TTAAGAAGGAAGATAGGCTCAGG
MeVIA2J8-594	1p1	16^e^	19	2.5	F: AACTTGGATAACTGGAAGAATGC R: GATGCCAATTAATCTGTGTTCC
MeVIA2M13-303	3q2-3 (1q3)^b^	73	115	2.5	F: GCGTACTACACAAGAAGGTGC R: GGTGTTACAGAATGTGCATAGG
MeVIA2M6-313	3q3 (5q1-2)^b^	87	107	2	F: AATCAACATGGTTCTTATTGTTCC R: CTCAAGGCGATGCTTATTCC
MeVIA2O13-302	1q3	74	123	2.5	F: GCGTACTACACAAGAAGGTGC R: GGTGTTACAGAATGTGCATAGG
MeVIA2P1-275	6p3	51	61	2.5	F: CATATGATAGAATAGGATGATTGGC R: TGTGACCAATAAGACCAGATAGG
MeVIA3B3-434	4p2.1	71	85	1.5	F: TTCATACAATTCCTCCATGCC R: AGAAGTTCAAGGTCACACAGC
MeVIA3C10-475	1q5	77	89	2.5	F: AAGTTAACAGAAGCAGACCTTGG R: AGTTCCATTCCAGCTTCACG
MeVIA3F20-234_LOWER	NA^d^	87	103	2.5	The same as MeVIA3F20-234_UPPER
MeVIA3F20-234_UPPER	2q2	90	92	2.5	F: CCTAGAAGAATCTGTTGCTGACC R: GCCTTATCTGTTGCAGAATCC
MeVIA3G11-104	7p3	75	117	2.5	F: TTAAGCATTAAGATTACATACATCTGC R: ATGGCGTGGTCTTCTTCC
MeVIA3G15-373	4q2 (1q2)^b^	63	67	1.5	F: TGAGAATGTCTCCTTCATGGC R: AATCCATAGTCTCTCTCTTGAGTCC
MeVIA3H17-399	3q2	79	80	2.5	F: CCATGTTATCTCCTGTCAATGC R: GTCACGAGCCAACTTCAGC
MeVIA3H22-576	1q4	63	63	2.5	F: GACCACATACAGAGAAGTACCTATGG R: CAGACTAAGTGCCATCTTCTGC
MeVIA3I8-498	4q1.1	94	122	1.5	F: GGCACATTCTCACCTCTACC R: TCTATGAGACCAAGAGCTTAATCC
MeVIA3L16-78	6q2-3	92	111	1.5	F: TAATCCATAAGGCCAGCTCC R: CATACAACTCATCAAGCTTCACC
MeVIA3M11-142	1q4-5 (2q3)^b^	91	99	1.5	F: TCTGATCACAGTGTCTCCTGC R: TTGTTGGTCATCGTATCTTCC
MeVIA3M4-293	3q3	89	87	2	F: ACATTCCAGCTTCTTCTGCC R: CCTCACACACACATATACATACACC
MeVIA3N11-345	2q1	93	118	2.5	F: TCGAGTCAGTATCACCAGCG R: TTAATACCTCCTCCATGCTCC

^a ^Set 2 markers are named as "BACName-Location", e.g. MeVIA3N11-344 is a marker within MeVIA BAC library clone 3N11, starting at base 344; while MeKba389E8-21049 is a marker within MeKba BAC library clone 389E8, starting at base 21049. In one case (BAC MeVIA3F20-234), two groups of PCR products were obtained from the single pair of primers, and each group was treated as a separate marker, named MeVIA3F20-234_UPPER and MeVIA3F20-234_LOWER.

^b ^The FISH-mapping locations of these markers are different from their linkage map locations (which are presented in brackets). Because of this conflict, these markers were not included in the final linkage map.

^c ^Secondary anonymous microsatellite locus that is co-amplified using these primers, and possibly could be a HPRT pseudogene.

^d ^NA = not available

^e ^Meiosis number less than 19, and not included in the map construction.

**Table 3:** Set 3 markers: reference gene, chromosome location and primer sequences

**Marker Name**^**a**^	Reference Gene	Chr	Arm	Band	Informative meioses		**Primer sequences**^**f**^
						
					M	F	
AM21	LFNG	3	p	2	90	84	F: TGCACTCCATGAAGACACTTG R: TCACTGGATTCAGATGGCTCT
ADCY1^d^	RAMP3	3	p	2.3	79	97	F: ACACATAGTCACTCTCCTTTACCG R: CAGAGAAGGGAGCCTGTTTAG
ASB7	ASB7	1	q	1	85	94	F: GGTCAGAGGACAACTAGGTTGAAG R: CATACAGAGGCAAAAGCATAACTG
C2orf54	SNED	6	q	3	80	64	F: TCCTCCAAATCCTCTTCCAGT R: CACTGCAAGCACCACTGTCT
C4orf8	TNIP2	6	p	3.1	56	95	F: CATGTCACCTGGAACTTTTTCA R: GTGTTGTATAGCTCAGTTTCAGATAGC
CACNG3	AQP8	1	p	1	88	112	F: AACTTTGGTGTCTTGGTGGAA R: TTTCAGTCACTGGGCTGAAGT
CASZ1	BCL3	5	q	3	42	79	F: AATGAGGGACAAGCAAGCTC R: AGTTGACCTCAGGGCAGTGT
CDH12	CDH12	4	p	2	75	96	F: TGCTACTACCCCATCTCTCTCTC R: CTTTCCAAAAGAACCAGAGCA
CLDN18	FAIM	5	q	2	62	89	F: GCAGAGCTGGCATTAGATGA R: TTTGTTCAATGACCCCCAAT
COL4A2	ARHGEF7	6	q	2	91	58	F: GAGAGGTCAGGGAAGGGTATCT R: TAAACCAGGTACTCCTGGGAAA
DLL1	USF1	2	q	3	94	93	F: ATAGGGAATGCAGCAGGTTG R: ATCAGCTGTTCTAAGGCCACA
DNHD2	TBX20	3	p	3	16^e^	70	F: CTGTCAAGTCTGAAGTGGACAGA R: GAGTTAATACTGGCGTCTTGGAG
EEF2K	KDELR2	3	p	2	83	76	F: AGGGCATCCCAAGATTCTTACT R: GCAGTGAAAATGACTAGGAGGAG
FIAM	FIAM	5	q	2	94	91	F: GCAATGCAAAGATGCACACT R: TGCTCCAGTGATGCCACTAC
GABBR2	QPRT	3	p	2	85	108	F: CTCCCAAGCTAGGAAACAACC R: CAAGACCGTATCAGAGGCAAA
HPX	CCKBR	5	q	2	86	69	F: GATCTCAGAAACATGGCCAGA R: CTGTACCCTCAAACCTTGTGC
IGF2R^b^	IGF2R	NA^c^	NA	NA	103	113	F: TACCTAGGTGGTTGACGCTGT R: AGACCTCACAAATTTGCCTTTC
MYCBP2	SLAIN1	6	q	2	71	42	F: CAGAGATTTTTGCCAGCAGAC R: CCCAACCTTTCAAGTAGAATGC
NOL14	NOL14	6	p	2	87	94	F: CCACCCCTCAGTGTTTCAGTAT R: GGTTAATGGGGCTTAGGATAGG
NRXN3^b^	NA	NA	NA	NA	83	98	F: GTTAGGGGCACAGCAGTGTAG R: CCGCAAGTCTTTTTAGCAATC
ODZ2	NUDCD2	1	q	2	81	77	F: AGCCCATAGTCAGGCACATAC R: GCACATAGAGGGAGTTGTCCA
PTCHD1	PTCHD1	5	p	2	94	111	F: TTTTTCTTCTCCCCCGTACC R: TGGCCTTGAAGCATACTTATTG
SFTPA1^b^	NA	NA	NA	NA	77	102	F: ACATGGGGGTAAAACTTGGAC R: TGAACCATGTCCTCTGACTCC
TBX4	FCRL4	2	q	3	88	88	F: TCACTCTATATCGGTCAGAGGACA R: GGTCTGGGACAGTAAATTCTTCAC
TCERG1L	TCERG1L	1	q	2	89	104	F: GACATATTAGCTGCTCTTCAGTGTTC R: GAGCTTGCTATGTCTGAAGGCTAC
TNFRSF11A	KDSR	4	p	2	101	110	F: TCTGTGTTCATTATCCGTGACA R: CATTGTGAGAAAGAGCCATCTG
TSHR	TSHR	7	q	1	97	97	F: TCTATGAGCCAAGAACTCCAGA R: GATGTTAGCAACAGAGATCATGGTA
TTHY	KIAA1012	4	q	1	78	76	F: CTCTTTCATTCCTAGACACACTGG R: GCAAGAAGAATGATGGACACAC
ZNF143	EIF4G2	6	p	3.2	92	90	F: GTTTATCACACCCAGGGACTGT R: GGTTAAGGTGCCAAAAGAGGTA

^a ^Set 3 markers are named after the gene within which they are found, e.g. IGF2R.

^b ^The reference genes for these three markers were planned for physical mapping, but not mapped at the time of primer design for markers. Therefore their physical locations were not known.

^c^Not available.

^d^ADCY1 is FISH mapped to 3p2.3, although its reference gene RAMP3 is mapped to 1p1.

^e ^Meiosis number less than 19, and not included in the map construction.

^f ^The MgCl_2 _PCR concentration for all set 3 markers is 2.5 mM.

### BAC libraries

Markers were derived from two tammar wallaby BAC clone libraries. The ME_VIA BAC library [9] was the first tammar wallaby BAC library, having 2.2× genome coverage; and the ME_KBa BAC library has 11× genome coverage (Arizona Genomics Institute, Tucson, AZ, USA, http://www.genome.arizona.edu/orders/ME_KBa_Clone).

### Markers and their physical locations

In order to achieve maximum coverage across the genome (i.e., ends of chromosomes and gap filling), and physically anchor and orient the linkage groups to chromosomes, three different approaches were used to identify sets of microsatellite markers for inclusion in the linkage map. The first approach identified 14 unique BAC-linked polymorphic microsatellites (set 1) discovered by screening 44 fluorescent *in-situ *hybridisation (FISH)-mapped tammar wallaby BACs, using an enrichment microsatellite screening technique according to Edwards et al. [10]. Since these BACs were already FISH-mapped, the physical location of each marker was taken as the location of its BAC. The second approach, yielding 47 polymorphic microsatellites (set 2), was to search BAC-end sequences for microsatellite repeat patterns using a custom Python script [11] as described by Macdonald et al. [12]. BAC-end sequences for the MeVIA library were generated by the Australian Genome Resource Facility. Sequences for the Me_Kba library were downloaded from Genbank [13,14]. The chromosomal location of each of these markers was determined by retrospectively FISH-mapping the corresponding BAC clones to tammar wallaby chromosomes [15]. The third approach produced 29 polymorphic microsatellites (set 3), which were chosen to fill gaps that became evident during the construction of the second-generation map. These markers were discovered by first identifying FISH-mapped genes that flanked gap regions. Using these FISH-mapped loci as reference genes, these gap regions were then aligned against the opossum genome sequence. Utilising the conserved genome relationship between the two species, genes that were predicted to fall within these gap regions in the tammar wallaby map were identified. These genes were then screened against the wallaby trace sequence archive (from 2 × genome sequence coverage) using BLAST software [16] to identify any orthologous wallaby gene sequence. All identified gene sequences were then assembled using CAP3 [17] to form a consensus gene contig. Finally, from these consensus sequences, 33 microsatellites were identified of which 29 were polymorphic. Consequently, the putative physical positions of these set-3 loci were set as the position of their reference genes. These set-3 markers were named after the genes within which the microsatellite sequences were found.

The full list of the above 90 new markers is presented in Tables 1, 2 and 3, together with related information. In addition, X-linked microsatellite markers (Mex34, Mex66 and Mex70) discovered by Macdonald et al. [18] were also included to supplement the X-chromosome linkage group.

### Genotyping

All 90 new microsatellite primers pairs were designed using Primer3 software [19] with the following settings: optimal primer length 21 bp (range 16-28), optimal Tm: 60°C (range 50-70°C), optimal GC content: 50% (range 30-70%), and amplification length: 100-600 bp. All other parameters were kept at default settings. Amplification of loci was performed via PCR on both male and female hybrid mapping pedigrees. The primer sequences and chromosomal positions for each of these loci are shown in Tables 1, 2 and 3. Each forward primer had an additional 19 bp of M13 sequence (5'-CACGACGTTGTAAAACGAC-3') added to the end to facilitate fluorescent labeling of products [20]. PCR was run on a PTC-100 DNA thermal cycler (MJ Research, Waltham, MA, USA) using a "60-to-50 touchdown" protocol according to Zenger et al. [7] incorporating 1.5,2.0 or 2.5 mM of MgCl**_2 _**(see Tables 1, 2 and 3) and 0.1 uM of each primer. Visualisation of PCR products was performed either using a LI-COR 4200 automated DNA sequencer or an ABI 3100 Genetic Analyzer (Applied Biosystems, Foster City, CA, USA). Genotypes were manually assigned and checked by two people independently to minimize genotyping scoring errors. Apart from between-run replicates (see below), all loci were genotyped across all individuals (i.e., male and female pedigrees) within the same genotyping run and platform (i.e. LICOR or ABI). If inconsistencies were observed in the data, additional genotyping was performed to resolve any problems.

### Data integrity

To ensure strict data integrity, two approaches described by Zenger et al. [7] were employed. The first approach incorporated the inclusion of duplicate samples both within and between each PCR/genotyping run for each locus. Within-run assessment was based on 9 replicate samples (~3% per PCR plate), while between-run assessment was based on the evaluation of 16 samples genotyped separately for each locus. Duplicate samples incorporated individuals from different pedigrees/generations with variable DNA qualities. The second approach used a custom program written in Perl to ensure strict Mendelian segregation of alleles within families across all three generations. Whenever an inconsistency was discovered, the original PCR results were examined for genotyping errors, which were corrected wherever possible. If no genotyping error could be detected, and the inconsistency remained unresolved, the relevant family data were removed from the data set.

To ensure strict concordance between the relative linkage map positions and the physical map positions, the assignment of each marker in each linkage group was examined in detail. Wherever the chromosome assignment of a marker conflicted with the chromosome assignment of the majority of markers in the same linkage group, its linkage and FISH-map locations were double checked by examining the PCR product sizes, sequencing the PCR products and (in most cases) repeating the FISH mapping. Any unresolved discrepant markers were removed before construction of the final linkage map.

### Segregation distortion

Segregation distortion in mapping loci can significantly compromise linkage map construction [21]. Segregation distortion is often observed in crosses between extremely inbred lines, or hybrids between divergent lineages [22,23]. To identify any such loci in the current study, segregation ratios in the male and female pedigrees were compared. When segregation patterns follow Mendelian inheritance, G2 offspring are expected to inherit equal numbers of grandsire and granddam alleles from the G1 hybrid individual. This expectation was tested via Chi-square analysis, using Benjamini-Hochberg's false discovery rate strategy [24] to allow for multiple testing (there were 95 markers in the female pedigrees and 83 markers in the male pedigrees).

### Map construction

Linkage maps were constructed using the software package CarthaGene 1.0 [25] which combines an EM (expectation-maximization) algorithm [26] and a local search technique in building a maximum likelihood map. Three maps were constructed: one from the female pedigrees (female map, i.e. from female meioses), one from the male pedigrees (male map, i.e. from male meioses), and one from the sex-pooled pedigrees (overall map). Input files for CarthaGene were automatically generated by a custom Perl program. This program assigns phase to the G2 genotypes based on G0 grandparent allele transmission through the G1 hybrid, and then formats the input files according to CarthaGene requirements. Linkage maps were constructed by grouping loci at a specific threshold and then ordering loci within each group at a specific confidence level.

Firstly, initial linkage groups were formed using the "group" command in CarthaGene, applying a minimum two-point LOD score (log of the odds score, which compares the likelihood of obtaining the test data if the two loci are indeed linked, to the likelihood of observing the same data purely by chance) threshold of 3.0 and a maximum recombination rate of 0.4. Any "orphan" marker that failed to be placed into a linkage group was further tested using a more powerful multipoint grouping approach. This approach calculates the likelihood of the odds supporting linkage between one locus and a framework order of loci, which utilises all available marker data to provide maximum power. Multipoint testing was performed using Mapmaker 3.0 [27] using the "try" command following generation of linkage group "framework" maps as described below. Any locus that displayed a significant multipoint association (LOD > 3) to a framework group was subsequently added to this group. Following this, any remaining orphan markers that had multipoint LOD scores approaching 3.0 and had also been FISH-mapped to the same chromosome as covered by a linkage group were also allocated to that same linkage group.

Once loci had been assigned to a linkage group on the basis of the LOD ≥ 3 criterion explained above, the second step of marker ordering within a linkage group was achieved by using three approaches with different levels of confidence (high, medium and low). A stringent marker order within each linkage group was first determined by constructing a framework linkage map containing only those loci that remained within a group after applying a threshold of LOD score of 3.0 (i.e., marker order fixed with log likelihood of next best map order < 0.001 probability) using the "buildfw" command in CarthaGene. The order of all the markers that appeared in these framework maps were rigidly fixed throughout the ordering processes of the remaining loci, and they were given the highest confidence level. Following this, a threshold LOD score of 2.0 was applied, enabling the ordering of further markers (next best map order < 0.01 probability), with a medium confidence level. Note that these markers had previously been allocated to this group on the basis of LOD ≥ 3. Once these had been positioned, they retained their order for the final assembly. The last step was to construct a maximum likelihood map with all remaining markers (i.e., not positioned in steps 1 and 2) within each linkage group, using the "build" command in CarthaGene, which constructs a comprehensive map, placing each remaining marker in its maximum likelihood position, followed by "polish" and "flips", which fine-tune the marker order. These markers, despite having been initially allocated to the group on the basis of LOD ≥ 3, were allocated the lowest confidence. The key point is that these low confidence markers were included without sacrificing the marker order in the framework map, which was fixed throughout the map-building process.

### Sex-specific differences

To evaluate sex-specific differences, a set of comparable male, female and sex-pooled maps was independently constructed using loci common to all three maps and in the same order. Using these comparable maps (not presented), sex-specific differences in recombination rates across pairwise marker intervals, chromosomes and the overall map were examined using an M-test and Chi-square heterogeneity tests on the LOD scores, according to Ott [28] and Zenger et al. [7].

### Genome Coverage

To indicate the extent of genome coverage, a FISH-mapped location was required for the first and the last markers in each linkage group. Where the end marker could not be reliably FISH-mapped (e.g. marker PB15 on chromosome MEU2p had insufficient sequence length for probe design), the next available marker (MeVIA3N11-345) was FISH-mapped instead. Five end-markers and their respective clones (G31-1, G16-2, T31-1, Y14-8 and PA55) identified from the first-generation map had insufficient sequence length required for FISH-mapping. Consequently, each of these loci had to be located within a BAC to gain additional sequence length. Each of these markers was first screened against the tammar wallaby ME_KBa BAC library for BACs containing these markers. Overgo probes for each marker were designed from sequence flanking the microsatellite, using the Overgo Maker program downloaded from Washington University Genome Sequencing Center http://genome.wustl.edu/software/overgo_maker. Overgo probes were radioactively labelled [29] and pooled for hybridisation. BACs isolated from this primary screen were subjected to a further round of screening via dot blots with individual probes, according to the protocol described by Deakin et al. [15].

The chromosome coverage of each linkage group was calculated as the average ratio of the length between the relevant pair of end markers to the total chromosome length, measured from 5 different metaphase spreads (10 chromosomes). In order to accurately locate the linkage map within each chromosome, the un-covered regions of each chromosome at the p telomere end and the q telomere end were also measured on the same spreads. Based on these results, the percentage of the uncovered p arm (named m%) and of the q arm (named n%) in each chromosome were calculated.

### Physical mapping by FISH

BACs containing end microsatellite markers for each linkage group were labelled by nick translation with Spectrum Green dUTP or SpectrumOrange dUTP (Abbott Molecular Inc., Des Plaines, IL, USA) and hybridised onto metaphase chromosomes following the protocol detailed by Alsop et al. [30]. Slides were washed following overnight hybridisation in 0.4 × SSC with 0.3% (v/v) Tween 20 for 2 minutes at 60°C, followed by a 1 minute wash at room temperature in 2 × SSC with 0.1% (v/v) Tween 20. Chromosomes were counterstained with DAPI (1.5 μg/ml) in Vectashield (Vector Laboratories Inc., Burlingame, CA, USA). Metaphase spreads and fluorescent signals were viewed using a Zeiss Axioplan2 epifluorescent microscope and captured on a SPOT RT Monochrome CCD camera (Diagnostic Instruments Inc, Sterling Heights, MI, USA) using IP Lab imaging software (Scanalytics Inc, Fairfax, VA, USA).

## Results

### Genotyping and data integrity

Genotyping of replicate individuals both within and between genotyping runs revealed a high level of concordance between samples (99.2% overall). The small number of discrepancies was primarily due to failure or low signal strength of poor quality replicate DNA. Inconsistency with Mendelian inheritance (i.e., offspring having an allele not present in either parent) was observed in 1.59% of animals in the female pedigrees and 0.71% of animals in the male pedigrees. Many of the observed Mendelian inconsistencies arise from a small number of loci in a select number of families. For example, three loci (KJW174, EEF2K & MeKba510M2-126695) in the female mapping pedigrees account for ~50% of the observed errors. Genotyping data were checked and corrected where possible; otherwise the data were excluded from the analysis. This resulted in the loss of 1.22% of the data overall.

Ten markers (MeVIA2C3-167, MeVIA3G15-373, MeKba510M2-pseudo, MeVIA2P1-275, ODZ2, C2orf54, MeVIA3L16-78, MYCBP2, MeVIA3H22-576 and NRXN3) in male pedigrees showed significant segregation distortion after correcting for multiple testing following Benjamini-Hochberg's strategy [24], and were subsequently removed from the male pedigrees data set (note: each locus is still retained in the female and sex-pooled maps, where available). No significant segregation distortion was found for any of the female map loci.

Seven markers (MeVIA3G15-373, MeKba281G14-77691, MeVIA2M13-303, MeVIA3M11-142, MeVIA2M6-313, MeVIA1G3-226 and MeKba273F23-57082) were removed from all maps (i.e., male, female and sex-pooled maps) after preliminary linkage map construction, because their FISH-mapped locations significantly deviated from their genetic linkage map position. This evaluation was based on the FISH locations of the vast majority of the markers in that linkage group. Also, one marker (CASZ1) presented irresolvable inconsistent results in the male and female maps, and was therefore also removed from the datasets for the final analyses. Finally, only those loci that had sufficient informative meiosis (≥ 19 based on power calculations [28]) were included in map construction. There were five autosomal markers in the male pedigree that did not satisfy this criterion and were removed prior to male map construction (see Tables 1, 2 and 3).

It is noted that females generally have a small increase in the numbers of informative meioses across loci. However, this is not unexpected as there are generally more G2 offspring available for this sex (i.e., 104 progeny from male pedigrees and 127 progeny from female pedigrees) and as such, there should be slightly more informative meiosis for female pedigrees when all the families are informative. For those 13 loci in the female pedigrees that display a slightly reduced number of informative meioses (see Tables 1, 2 and 3), this is a direct result of a small number of female families being non-informative (i.e., female G1 individual homozygous). Furthermore, it appears coincidental that 10 of these 13 loci are from marker set 3 (Table 3).

### Map construction

**Figure 1:**
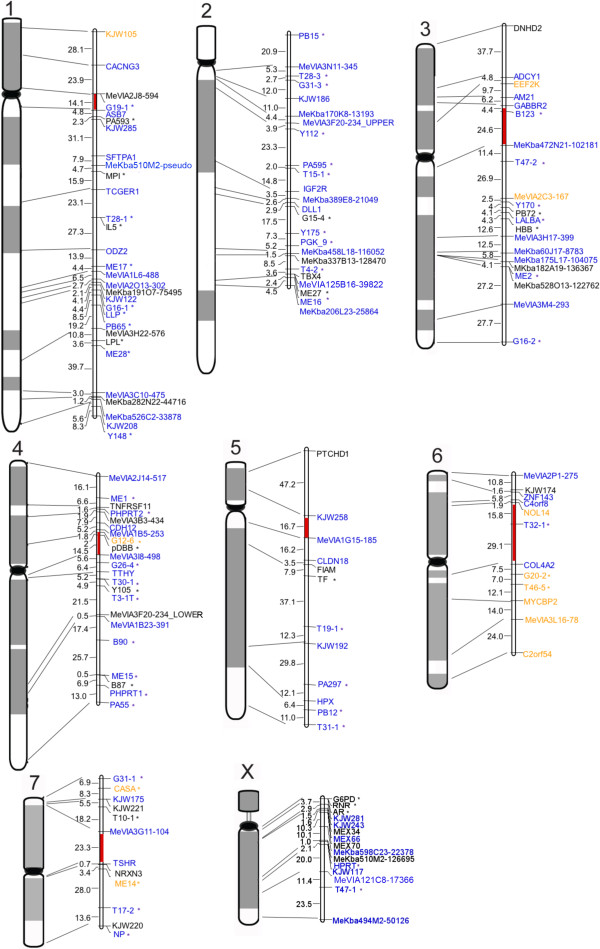
**The second-generation sex-pooled tammar wallaby linkage map and corresponding cytogenetic map**. For each chromosome, the cytogenetic map is presented on the left and linkage map on the right, with lines connecting markers mapped in both maps. All linkage maps are presented with the p telomere end at the top and the q telomere end at the bottom. Centromere locations were estimated from FISH-mapping data and are labelled in red, except for MEU2 and MEUX, where the linkage maps cover only the q arm of the chromosomes. Centimorgan distances between flanking markers are listed on the left side of each map.. Markers are shown in different colours to distinguish their location confidence levels: blue for high level of confidence where it is 1000:1 odds to next possible location; orange for medium level of confidence where it is 100:1 odds to next possible location; black for low level of confidence where it is the most likely location. All first generation markers are labelled with a "*" mark.

The final sex-pooled (overall) linkage map consists of 148 markers comprising 84 second-generation markers and 64 first-generation markers (Figure 1). This map is accessible online at http://compldb.angis.org.au/. Based on both two-point and multipoint groupings at LOD threshold of 3.0, 146 loci formed eight linkage groups (one per chromosome, i.e. MEU1 to MEU7, and MEUX). In total, three orphan markers (MeVIA1A16-106, DNHD2 and PTCHD1) could not be placed into any linkage groups based on the LOD threshold ≥ 3. However, two of these orphan markers (DNHD2 and PTCHD1) were eventually placed in their respective linkage groups (at low confidence) based on FISH-mapped locations and respective multipoint LOD scores of 2.32 and 2.86, respectively. In the final stages of constructing the overall map, there were 99 markers with high confidence, 12 with medium confidence and 37 with low confidence. With more than two-thirds of markers assigned a map position at the highest confidence level (i.e., framework linkage map) and average marker interval distance of 10.9 cM, the number of informative loci and number of individuals genotyped was appropriate for developing a suitable genome-wide framework linkage map.

As shown in Additional File 1, the total sex-pooled map size is 1402.4 cM, which extends the first-generation map length by more than 40%. Within this map, there are 33 loci (including 15 loci from X chromosome) based on female meiosis data only, and 12 based on male meiosis data only. There are 136 and 115 loci in the female and male map respectively. The overall and sex-specific linkage maps including allele number data are presented in tabular format in Additional File 1.

### Sex-specific differences

As shown in Table 4, 11 of 96 (11%) intervals common to male and female maps displayed significant difference (p < 0.05) in recombination rates. Five of these 11 intervals present higher female recombination rate while the other six intervals demonstrate higher male recombination rate. At the chromosome level, three of seven chromosomes (MEU1, MEU3 and MEU6) exhibited significant differences between sexes (p < 0.05), all showing a higher male recombination rate. With the overall comparable (i.e. built with the same set of markers) female and male map sizes of 1066.5 cM and 1272.2 cM respectively; the F/M ratio is 0.84, which is significantly different (p < 0.001). In total, marker interval regions displaying significant sex-recombination differences are distributed on six chromosomes, covering 8.8% of the entire comparable map length (i.e. the length of the map built up with common markers in both sexes), and the three chromosomes displaying overall significant sex-recombination differences add up to 48.2% of the total comparable map length.

**Table 4:** Intervals exhibiting significantly different sex-specific recombination rates

Chr	Interval		Sig.^a^	Co-informative meiosis		F/M ratio	Female (cM)	Male (cM)
						
	First marker	Last marker		F	M			
1	MeVIA1L6-488	ME17	**	112	80	0.09	0.9	9.6

1	ME17	T28-1	*	72	71	0.39	22.6	58.5

1	T28-1	TCGER1	*	67	75	0.37	13.7	37

1	Whole chromosome		**			0.72	246.5	341.3

2	T15-1	PA595	**	121	90	0	0	4.4

3	B123	MeKba472N21-102181	*	81	77	1.79	31.2	17.4

3	MeKba182A19-136367	ME2	*	81	91	0	0	4.9

3	Whole chromosome		*			0.97	195.2	202.2

4	TTHY	G26-4	*	76	75	3.36	9.4	2.8

4	MeVIA3I8-498	MeVIA1B5-253	*	101	82	2.02	21.6	10.7

6	NOL14	C4orf8	*	77	51	0.43	1.2	2.8

6	ZNF143	KJW174	*	74	89	NA^b^	3.4	0

6	Whole chromosome		*			0.85	63.8	75.3

7	T10-1	KJW221	*	120	77	5.71	8	1.4

Overall			***			0.84	1066.5	1272.2

**^a^**There are three significant levels: *, 0.01 < p < 0.05; **, 0.001 < p < 0.01; ***, p < 0.001.

**^b ^**NA = data not available, as the divider is zero.

There was no consistent pattern in either male or female map intervals exhibiting sex-specific recombination differences. On MEU6 the interval with larger male map sizes was located closer to the centromere than the interval with larger female map size, whereas on MEU3 the interval with larger female map length is closer to the centromere. MEU4 contains two intervals both with larger female map size close to the centromere, MEU7 contains an interval with a larger female map size in a medial position, and MEU1 and MEU2 contain intervals with larger male map size in the middle of a chromosome arm.

### Genome coverage

**Figure 2:**
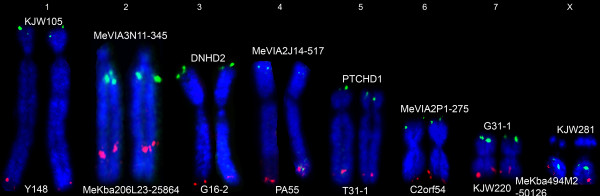
**Linkage map coverage (two-colour FISH map of end-markers for each linkage group)**. Localisation of BAC clones containing markers for the ends of each linkage group on tammar wallaby metaphase chromosomes, with p arm at top and q arm at bottom; marker names labelled at two ends of each chromosome and for details of markers see Table 5.

The genome coverage of the overall map is illustrated in Figure 2. The genome is well covered by the linkage map, except for the short arms of MEU2 and MEUX, and a distal region of MEU2q. Details of the FISH-mapped end (or near-end) markers, their BAC clone information and the coverage measurements are presented in Table 5. The presence of a relatively large nucleolus organiser region (NOR) on the short arm of chromosome X (Xp), which is differentially contracted on the inactive X of females [31], could bias the result [32], so Xp was excluded from the following estimates. As can be seen in Table 5, the estimated total genome size (assuming uniform cM/physical distance and allowing for exclusion of Xp), is 1698.2 cM. Given that the total length of the second-generation linkage map is 1402.4 cM, this gives an estimated genome coverage of 82.6% without Xp being taken into account. The percentage of uncovered chromosome regions at the p telomere and q telomere ends (named m% and n%, respectively) in each chromosome was calculated and is shown in Table 5.

**Table 5:** Linkage map end-markers used in genome coverage estimation, their BAC clones and overgo sequences, probe colours, cM and percentage coverage of the chromosome.

Chr	End marker	BAC clone	Overgo sequences	Probe colour (Fig. 1)	cM length covered by end markers FISH-mapped in Fig. 2	% of chr included	chr length (cM)	**m%**^**c**^	**n%**^**d**^
	KJW105	MeVIA_123J11	NA^a^	Green	321.2	93.76	342.6	3.51	2.73
						
1	Y148	MeKba_448F6	a: CCAAACTGTAATGAAGGTTCAATG b: GCAGGTTACTTAGCTACATTGAAC	Red					

2	MeVIA3N11-345	MeVIA_3N11	NA	Green	138.9	56.24	247.0	18.21	25.55
						
	MeKba206L23-25864	MeKba_206L23	NA	Red					

3	DNHD2	MeKba_175F3	NA	Green	230.5	95.09	242.4	3.18	1.73
						
	G16-2	MeKba_323D15	a: GAGTTCAAATCCAGTCTCTCTTAC b: CAGGGTTGCATAGTGAGTAAGAGA	Red					

4	MeVIA2J14-517	MeVIA_2J14	NA	Green	165.2	88.93	185.8	9.89	1.18
						
	PA55	MeKba_369C8	a: TCTACAAAATGAGGATAATACTCC b: GAACCCCTGTGAAGTAGGAGTATT	Red					

5	PTCHD1	MeKba_555M23	NA	Green	200.2	86.04	232.7	1.89	12.16
						
	T31-1	MeKba_345M2	a: CTGGGATTCTAAGAGGGTGAGAAG b: TCCCCAAATCCTGGTTCTTCTCAC	Red					

6	MeVIA2P1-275	MeVIA_2P1	NA	Green	129.6	96.58	134.2	2.72	0.69
						
	C2orf54	MeKba_307P14	Na	Red					

7	G31-1	MeKba_80D8	a: TATTTCCCTAGGGAGGGACATCTG b: AGGAAGATGGTGCTTGCAGATGTC	Green	107.9	87.17	123.8	1.38	11.45
						
	KJW220	MeVIA_115N20	NA	Red					

X	KJW281	MeVIA_15A6	NA	Green	88.1	25.84 (46.43**^b^**)	189.7**^b^**	70.36	3.81
						
	MeKba494M2-50126	MeKba_494M2	NA	Red					

			TOTALS		1381.6		1698.2		

The final column shows the estimated full chromosome lengths, calculated from the previous two columns.

^a ^NA = not available, as no overgo probes were designed for this marker.

^b ^The value 46.43 is the estimated % coverage of Xq and 189.7 is the estimated cM size of Xq, as the 25.84% physical size coverage of MEUX may not reflect the linkage length coverage because of a relative large nucleolus organiser region (NOR) on Xp.

^c ^m% = the percentage of the length of the p arm not covered by the linkage map, for a particular chromosome.

^d ^n% = the percentage of the length of the q arm not covered by the linkage map, for a particular chromosome.

## Discussion

### Marker selection

The approaches of identifying novel microsatellite markers within BAC clones, BAC-end sequences and through a comparative genomics approach for gap filling were extremely successful. The first approach delivered 14 unique BAC-linked polymorphic markers from only 44 BACs, the second approach yielded 47 unique BAC-linked polymorphic markers from partial BAC end-sequence data, while the third, comparative-genomics, approach revealed 29 polymorphic gene-specific microsatellite loci. The identification and physical positioning of microsatellite markers using these different approaches was very productive.

Interestingly, this mapping study has confirmed an evolutionary breakpoint between two closely positioned markers ADCY1 and RAMP3 on MDO6 (*Monodelphis domestica *chromosome 6, which is homologous to MEU1 and MEU3). ADCY1 failed to be positioned alongside RAMP3 on MEU1p1 according to its initial predicted comparative position in *M. domestica *(ADCY1 and RAMP3 are also co-located together in eutherian genomes, e.g., human and bovine). Instead, it was mapped to MEU3 by both linkage and FISH mapping techniques. Based on physically mapped flanking loci, ADCY1 is in close proximity to an evolutionary breakpoint (Wang et al., submitted). We believe that ADCY1 is separated from RAMP3 due to this evolutionary breakpoint, which is also supported by the mapping data of its flanking loci on MEU3.

Our comparative approach to identifying loci and developing markers to fill gap regions significantly accelerated the mapping process in this study, and reduced the total number of loci needed to achieve the same genome coverage. Reliance on only anonymous random markers would have required at least 35% more markers to obtain the same level of genome coverage at 99% power [28].

### Discrepant marker positions, Mendelian inconsistency and segregation distortion

One of the main objectives of this study is to produce a robust anchored genetic linkage map incorporating both physical and genetic linkage mapping data. Consequently, seven markers were removed from the final linkage map due to major discrepancies between the linkage and BAC FISH locations. Neither the linkage map nor the physical map position could be confirmed as the true map position for any of these loci. One possible explanation is that the microsatellite primer pairs amplified a secondary product other than target sequence from which they were derived (e.g., locus duplication). Alternatively, a BAC-clone identification error could have occurred during the FISH mapping process, or the BAC-end sequences stored in GenBank could be listed with incorrect BAC names. After removal of these seven loci, there is excellent agreement between linkage and FISH maps (Figure 1). Only one locus shows slight differences (EEF2K), but this is due to a reduced confidence mapping position in the linkage map rather than true differences.

The overall level of Mendelian inconsistencies observed in the genotype data (1.22%) is relatively low considering that the loci used are novel without any prior information. The majority of these inconsistencies arise from a small number of loci in a select number of families. For example, three loci (KJW174, EEF2K & MeKba510M2-126695) in the female mapping pedigrees account for almost 50% of the observed female pedigree error rate (1.59%). It would appear that null-alleles and/or allelic drop-outs are present within these loci. Ignoring these three loci, the observed female pedigree error rate reduces to 0.81%, which is comparable to that of the male pedigrees (0.71%). The remainder of observed errors can be attributed to poor quality DNA in a small number of individuals, a few difficult-to-score loci and several *de novo *germ-line mutations. In all situations, non-Mendelian genotypes (i.e., specific family data) were removed from the dataset. We discount the possibility of pedigree errors because this resource has been rigorously tested over a period of 20 years across multiple projects; and there were no families in which the Mendelian inconsistency pattern was consistent across all informative loci. All anomalies were individually inspected using our custom Perl script, and no animal displayed evidence of an aberrant error rate indicating incorrect assignment. We concede that in many highly-studied organisms (e.g. human and mice) one might see somewhat lower inconsistency rates, but this is expected, given the level of information available for standardized mapping loci sets in highly-studied species.

Ten loci in the male pedigrees showed significant transmission segregation distortions (p < 0.05). However, after close inspection of genotypes, it was determined that the distortion was caused by allele assignment bias derived from non-informative genotypes rather than true biological segregation distortion (e.g., post-zygotic selection). This effect was localised only to male pedigrees (due to genotype and pedigree composition) and as such these segregation distortions were not observed for female pedigrees. Although the segregation distortion was a result of non-informative pedigree individuals, the inclusion of these markers in the male pedigree map construction could have still caused unpredictable problems in analysis, so these loci were excluded from the map constructions.

In situations where a locus has been identified as a possible discordant marker and not totally excluded from both male and female pedigrees, these loci were tested in the remaining pedigree to determine if they adversely affected linkage map length. For both the three loci that displayed non-Mendelian inheritance in specific families and the ten segregation-distorted loci, linkage map building was conducted with and without these markers. In all situations, the inclusion of these loci has no adverse effect on map length (0.25-3.7% difference), and as such they were retained in the remaining pedigrees.

### Linkage groups

Linkage map construction produced eight linkage groups that correspond to and cover large portions of the eight chromosomes of the female tammar wallaby (autosomes MEU1 to MEU7, and the X chromosome MEUX). Final orientation of linkage groups on chromosomes was determined from both linkage group data and physical FISH mapping information (Figure 1). The non-recombining Y chromosome (MEUY) is not represented by linkage data so is not considered in this study. However, there have been ten microsatellite markers reported in MEUY [12], which could complement our linkage map once these Y chromosome loci have been confidently positioned using other methods.

The second-generation linkage map is a substantial improvement upon the first-generation map in terms of number of loci mapped, genome coverage and physical placement of loci/linkage groups on the chromosomes. The number of loci in this current map (n = 148) more than doubles the number from the first-generation map (n = 64). The coverage of the genome has also significantly improved, with the total map size increasing from 828.4 cM to 1402.4 cM, and the predicted genome coverage from 42.8% to 82.6%. The average map distance between adjacent markers was decreased from 16.2 cM to 10.9 cM. A chromosome-by-chromosome comparison of the two maps is presented in Additional File 2. The assignment of linkage group 2 (LG2) to chromosome 1 in the first-generation map has now been corrected by its relocation to chromosome 4. The assignment of this linkage group was originally based on the physical position of a DBB-like clone, which has since been shown to be a pseudogene, here renamed pDBB in this second-generation map.

### Recombination Rate Female Vs. Male

The overall sex-specific difference in recombination rates in this study is relatively similar to that from the first-generation map (0.84 and 0.78, respectively). This pattern was inconsistent across the genome (Table 4) and there is no evident bias in chromosome position of intervals with higher male or female recombination. In eutherian mammals, the heterogametic sex (i.e., males) typically shows lower recombination rates [33-36], but in marsupials the reverse pattern has been reported. The first reported linkage dataset of an Australian marsupial species, *Sminthopsis crassicaudata*, revealed large differences between female and male recombination rate with less recombination in female [37], and preferential positioning of chiasmata close to telomeres in female meioses and "interstitial" in male meioses. Similar results were obtained from a study of chiasmata positioning in the South American gray short-tailed opossum (*Monodelphis domestica*), and a severely reduced female recombination rate was later reported [38-41], suggesting that this sex difference in chiasmata distribution might present in all marsupials [42]. Thereafter sex differences in chiasmata distribution and recombination rate have been noted in several linkage studies in marsupials with no simple pattern being established. In another Australian marsupial, the brush-tailed possum, *Trichosurus vulpecula*, chiasma number was lower in female meiosis, though not so dramatically [43] and the chiasmata distribution was not significantly different between the sexes. The western brushed-tail bettong *Bettongia penicillata*, a species from a distantly related Australian marsupial group, showed no obvious difference in chiasma number and localisation between sexes [44].

Inconsistent sex-recombination results have been reported for the tammar wallaby (*Macropus eugenii*). An early study found higher female recombination rate for two pairs of markers [45], whereas the first comprehensive linkage mapping study revealed a reduced female map size similar to other marsupials [7]. These inconsistencies are accounted for by our present findings that different intervals on different chromosomes show greater male or female recombination rates, and that there is no consistent pattern of bias over chromosome arms. A dense linkage map with smaller marker intervals will be needed to provide a more complete description of the recombination rate difference between sexes in this species.

### Applications and future direction

The microsatellite markers discovered in this study have the potential of being applied in other macropod species, as marker transferability has been shown to be relatively high among macropodoid taxa (average ~65%) [12,46]. The anchored genetic linkage map of *M. eugenii *provides a valuable resource, not only for comparative mapping purposes and positional cloning, but also as a bridging framework scaffold for assisting with assembly of the tammar wallaby genome sequence assembly. This new map has been used to create a virtual tammar wallaby genome map (Wang et al. submitted), which will serve as a backbone for the genome sequence assembly. This map and available mapping pedigree resources also provide a solid foundation for future high-density mapping studies, incorporating tens of thousands of genome-wide SNP markers, and the complete physical anchoring of these SNPs/genes to the tammar wallaby genome assembly using modern high-throughput genotyping and mapping techniques.

## Conclusions

A second-generation anchored tammar wallaby linkage map with 82.6% genome coverage was constructed with 148 markers, using both linkage and FISH-mapping data. This map will be a valuable tool for gene localization and comparative studies. When combined with the full cohort of available physical mapping data, sequence data and comparative data, this mapping resource will significantly contribute to the better understanding of marsupial genome structure, function and evolution. It has already been instrumental in the construction of an integrated and virtual tammar wallaby genome map (Wang et al. submitted), which provides a backbone for the 2-fold tammar wallaby genome sequence assembly [3].

## Authors' contributions

CW was primarily responsible for data compilation, data analysis and preparation of the manuscript and was heavily involved in genotyping especially on the LICOR system; KJW produced set-1 markers; MJW discovered set-2 markers; HRP discovered set-3 markers; LW generated genotype data; JED and AA FISH-mapped markers to the genome; JED screened the BAC libraries for end markers and assisted in the genome coverage analysis; KRZ was responsible for project design, generated genotype data, assisted with analysis and revised the manuscript; FWN worked closely with CW during the analysis and writing phases; DWC provided the animal DNA resources; JAMG provided funding, devised strategies for set-1 and set-3 marker discovery, and revised the manuscript. All authors read and approved the final manuscript.

## Supplementary Material

Additional file 1 **Linkage maps**. The overall, female and male linkage maps in tabular format, with additional information about the number of informative meioses and the number of alleles for each marker. Click here for file

Additional file 2 **Comparison with the first-generation linkage map**. A chromosome-by-chromosome comparison with the first-generation linkage map regarding the number of markers and map lengths. Click here for file
